# Interfaces of occupational health management and corporate social responsibility: a multi-centre qualitative study from Germany

**DOI:** 10.1186/s12889-021-11016-z

**Published:** 2021-06-02

**Authors:** Eva Kuhn, Sebastian Müller, Christoph Teusch, Grit Tanner, Marlies Schümann, Carolin Baur, Eva Bamberg, Ludger Heidbrink, Stuart McLennan, Alena Buyx

**Affiliations:** 1 Institute of History and Ethics in Medicine, Technical University of Munich, Ismaninger Str. 22, 81675 Munich, Germany; 2 Chair of Practical Philosophy, Kiel University, Leibnizstr. 4, 24118 Kiel, Germany; 3 Die VERBRAUCHER INITIATIVE e.V. (Bundesverband), Berliner Allee 105, 13088 Berlin, Germany; 4 Department of Work and Organizational Psychology, Beuth University of Applied Sciences Berlin, Lütticher Straße 38, 13353 Berlin, Germany; 5 Leadership Excellence Institute Zeppelin, Zeppelin University, Am Seemooser Horn 20, 88045 Friedrichshafen, Germany; 6 Work and Organizational Psychology, University of Hamburg, Von-Melle-Park 5, 20146 Hamburg, Germany; 7 Institute for Biomedical Ethics, University of Basel, Bernoullistrasse 28, 4056 Basel, Switzerland

**Keywords:** Workplace health promotion, Corporate philosophy, Ethical values, Company culture, Germany

## Abstract

**Background:**

The workplace has been identified as a priority setting for health promotion. There are potential advantages of systematically integrating Occupational Health Management (OHM) and Corporate Social Responsibility (CSR). However, OHM and CSR are usually overseen by different management branches with different sets of values, and there is a lack of empirical research regarding interfaces between OHM and CSR. Germany offers a particularly useful setting due to legislation requiring health to be promoted in the workplace. This study aims to examine key stakeholders’ views and experiences regarding interfaces between OHM and CSR in German companies.

**Methods:**

Individual semi-structured qualitative interviews were conducted with a sample of 77 German stakeholders from three different groups: experts in occupational health and corporate social responsibility from various companies (*n* = 35), business partners (*n* = 19), and various non-business partners (*n* = 23). Transcripts were analysed using qualitative content analysis.

**Results:**

Participants identified several areas in which OHM and CSR are already interacting at strategic, structural and cultural levels, but also highlighted several barriers that undermine a more meaningful interaction. Participants reported difficulties in articulating the underlying ethical values relevant to both OHM and CSR at the strategic level. Several structural barriers were also highlighted, including a lack of resources (both financial and knowledge), and OHM and CSR departments not being fully developed or undertaken at entirely different operational levels. Finally, the missing practical implementation of corporate philosophy was identified as a critical cultural barrier to interfaces between OHM and CSR, with existing guidelines and companies’ philosophies that already connect OHM and CSR not being embraced by employees and managers.

**Conclusions:**

There is already significant overlap in the focus of OHM and CSR, at the structural, strategic and cultural levels in many German companies. The potential is there, both in theory and practice, for the systematic combination of OHM and CSR. The insights from this study will be useful to ensure that closer integration between both management branches is set up in a socially sustainable and ethical manner.

**Supplementary Information:**

The online version contains supplementary material available at 10.1186/s12889-021-11016-z.

## Background

Workplace health promotion (WHP) includes “the combined efforts of employers, employees and society to improve the health and well-being of people at work” [1]. WHP and particularly behaviour-based prevention such as physical activity [2, 3] is one significant part of Occupational Health Management (OHM). Although no universally accepted definition of OHM exists, it typically includes legally mandated occupational health and safety, behaviour-oriented measures aimed at promoting well-being and health by encouraging individual employees to change their behaviour, and system-oriented measures aimed at improving working conditions and thereby contributing to a healthy workplace for all employees [4, 5]. A potentially relevant management framework for OHM is Corporate Social Responsibility (CSR), which focuses on reasons for engaging in activities that transcend a company’s core financial goals [6]. Although employee health and safety were not traditionally a central element of CSR, some important overlaps already exist. In the last years, research and business practice outside Europe has put particular emphasis on the integration of occupational health and safety (OHS) reporting and measures in CSR, focussing on occupational illnesses, work-related injuries, accidents, and sick days [7–9]. Others tackle the link between CSR and the social determinants of health both for internal and external stakeholders [10, 11]. However, WHP and OHM are company-internal offers that go beyond OHS. So far, they are only rarely considered to be part of CSR, but this view is increasingly being reconsidered on both the political and scientific levels [6, 12–15].

The legal framework in Germany regarding WHP provides an interesting example of the increasing overlap between OHM and CSR. With the enactment of the Prevention Act in 2015, WHP became part of German social legislation. Health insurance providers are now required to promote health in the workplace, in addition to their previous duties under the statutory accident insurance and the Occupational Safety Act, to assist the implementation of this new legislation. German health care insurers offer consultation services on how to design and implement OH measures for companies, various health promotion and prevention packages for direct implementation, and financial support to companies [16]. In addition, other direct financial incentives for companies are available, e.g. via the Income Tax Law.

Existing research in Germany indicates that three in ten German companies offer a range of individual-oriented interventions [17]. It has also been found that in-house personnel from various departments (including human resources, strategy, sustainability, public relations, CSR etc.) are increasingly engaging in OHM activities [18]. Industry and trade associations and various public institutions are also increasingly developing guidance and information for companies on WHP and OHM [19]. Moreover, it is common in large companies for employees to establish so-called “health circles”, where topics such as smoking cessation and work-life-balance are covered; providing an important intersection between management and employees [20]. In contrast, small and medium-sized enterprises rarely have their own in-house OHM systems, but can receive external support from public institutions or private providers [21].

Beyond regulatory compliance, it has been speculated that systematically linking OHM and CSR may potentially have several competitive advantages for companies; such as an enhanced corporate image and reputation among their consumers, employees, and business partners, and savings due to intra-organisational synergies [14, 22–24]. In the long term, this has the potential to ultimately strengthen a company’s market position [25, 26], and may also have wider social or ethical benefits, if they address organisational, communal or global justice issues [27, 28]. Synthesizing voluntary CSR and partly mandatory OHM may also help companies comply best with their social and ethical responsibility for the health and safety of their employees [23, 29].

CSR and OHM, however, are usually overseen by different management branches and based on different sets of values [30]. There is a lack of empirical research regarding the current interfaces between OHM and CSR in companies and how stakeholders perceive the integration of the two management branches. Due to the enactment of legislation requiring health to be promoted in the workplace, Germany’s system offers a particularly useful setting to examine these issues. The aim of this study, therefore, is to examine key stakeholders’ views and experience regarding interfaces between OHM and CSR in German companies. A comprehensive overview of these issues will help identify the key facilitators and barriers to implementing a more systematic approach to synthesising OHM and CSR.

## Methods

The methods used in the study are presented below, following the ‘Consolidated criteria for reporting qualitative research’ (COREQ) [31]. Ethics approval was waived by the Local Ethics Committee of the Faculty of Psychology and Human Movement Science at the University of Hamburg.

### Research team and reflexivity

Individual interviews were conducted by EK, SM, CT, GT or CB, who all had previous training and experience in qualitative research. No relationship was established between the interviewers and the participants prior to the study, and participants received limited information about the researchers. No hierarchical relationship existed between the researchers and the study participants.

### Study design

The theoretical framework employed in this study was qualitative content analysis [32, 33]. Participants were primarily selected through purposive sampling, to ensure sample diversity according to predetermined factors (e.g., industry representation, company size, established reputation regarding OHM and/or CSR, etc.). Participants were contacted by e-mail and suitable interview dates were determined for those willing to participate. Seventy-seven participants agreed to participate in the study and were recruited from three different groups (see Table 1): OHM and/or CSR experts from various companies of different sizes and industry sectors (*n* = 35), business partners such as suppliers and contractors (*n* = 19) and various non-business partners, namely insurances, public institutions and employee representations (*n* = 23).

**Table 1:** Overview of the experts interviewed

Category	Interview guide	Interviews
**Companies**	OH	18
	CSR	10
	both	7
**Business partners**	OH	5
	CSR	3
	both	11
**Non-business partners**	OH	5 health/ social insurances
		1 public institution
		3 employee representations (works council or trade unions)
		3 Other (NGOs, employers’ associations, etc.)
	CSR	0
	both	2 health/ social insurances
		7 public institutions
		2 employee representations (works council or trade unions)

Semi-structured interview guides (OHM and CSR) were developed by the authors (see Additional File 1). Interview guides were pre-tested twice and discussed among the research consortium to minimise the risk of personal preconceptions [34]. Interviews were held between November 2016 to September 2017. Fifty-six interviews were conducted in person at a venue of the participants’ choosing, while the remaining 21 interviews were conducted via telephone. Seventy-six interviews were conducted in German, and one interview was conducted in English. Only the participant and the researcher were present during the interview. No repeat interviews were carried out. Only one participant did not consent to the interview being audio recorded. Participants were informed that the interviews would be immediately pseudonymised and later fully anonymised [35]. Field notes were taken after the interview that had not been audio recorded. After 77 interviews, data saturation arose, and the research team concluded that saturation had been reached with regard to the content and attitudes expressed by the participants [36]. Transcripts of the interviews were returned to participants for approval.

### Analysis and findings

Interview transcripts were analysed in their original language using qualitative content analysis by at least two coders with the software MAXQDA [32, 33]. The analysis began with setting up a first coding frame in a deductive manner by drawing on former research on the interfaces of OHM and CSR. Line-by-line coding was carried out with all interviews and the relevant meaning of each code was made explicit by either assigning it to an existing category or creating a new category. During this step, initial themes identified as common across participants as well as those unique to individuals were labelled. These findings were integrated into the coding frame, resulting in a deductive-inductive approach to the interview material. The coding frame was discussed within the research group to establish intercoder reliability and to resolve coding differences [37]. Findings are presented as higher- and lower-level categories in the coding frame. The analysis, displayed by the growing coding frame, revealed that the themes identified can be further categorised under the three ordering moments of The New St. Gallen Management Model: strategy, structure and culture [38]. Hence, this structure was added deductively to the coding frame at a later stage. Finally, a communicative validation was conducted during an expert workshop in which ten interview participants and seven other experts participated [33]. The workshop participants were permitted to remain anonymous so that they could comfortably share their experiences of ‘worst practices’ and critical moments regarding OHM. The potential results of a relationship between OHM and CSR were discussed and slightly adjusted afterwards, without the need to edit the coding frame. Selected quotes for English language publication were translated separately by EK and SM and results were compared to maximise intercoder reliability. Following the method of van Nes and colleagues, validity was ensured through the use of translations that adhered closely to the original sentence structure and by focusing on the quantity as well as the type of words used by the participants; therefore, grammatical and other errors have not been corrected [39].

## Results

A total of 13 distinct sub-categories regarding the relationship between OHM and CSR in German companies were identified, consisting of seven existing or planned interfaces and six barriers to or reasons against interfaces between the two. Table 2 gives a full and detailed account of issues identified, along with example quotes. The definitions and coding rules used in the coding framework are presented in the Appendix.

**Table 2:** Result of the Qualitative Content Analysis for OHM-CSR interfaces and concerns mentioned

Node	Main Category	Subcategory	Text example (direct quotes)
**Existing interfaces & Interfaces that should be established**	Structural	Joint actions	[…] a corporate run and kilometres. Then, the business gives something for social projects (CB213, organisation, department director).
		Overlap of responsibilities a) in the same department b) for the same person	The person responsible for OHM reports to my colleague who is responsible for ‘sustainability’ in the other business unit. (VU04, company, head of Corporate Responsibility (CR) and Sustainability)
		Extension of the health circle or CSR-board	Sure, someone from human resources also takes part in our task force ‘sustainability’. (VU08, company, responsible person for sustainability management).
	Strategic	Standards and certification	[…] the sustainability codex […] says that we […] have a global, holistic Health Management (HU08, company, OHM representative)
		In general	[…] if one wants to have the external image of an absolutely ‘clean’, superb company, then Occupational Health Management in my view belongs right at the front. (CS22, business partner, human resource manager)
	Cultural	Corporate philosophy	However, in any case a company’s or company manager’s catalogue of values has an influence on health again and again […] (CB212c, institution, representative of a statutory health insurance)
		Health as part of the leading principle “social responsibility‘	If there is any opportunity at all to sensitise large groups [in that case: millions of employees in Germany] for the subject of ‘health’, then only in the workplace (HU40, company, OHM representative; cf. HB41, business partner, not specified).
**Open concerns & Reasons for no existing/ planned interface**	Structural	Lack of resources	[CSR and OHM] are different communities and there one has to establish a platform, and this costs money. I mean, time is money. (CB210, government institution, occupational safety)
		No appropriate internal structures and/ or knowledge in the company	So, for us in a first step it actually is about […] creating structures by means of which we also can systematically take care of the health issues of our employees in the future. (HU39, company, OHM representative)
		Location of OHM and CSR at different operational levels	CSR is managed from the United States [WHP is based in Germany]. (CS25, business partner, head of human resources)
	Strategic	Lack of overlapping stakeholders	Health Management and Promotion often concerns the own employees and their families, but CSR addresses a larger group. (VB07, organisation, responsible person for Social Security)
		OHM not primarily a company task	[Such an interface] must be implemented on a macrosocial level. […] A frame must be provided that enables companies [to take up responsibility in CSR and OHM]. (VB07, organisation, responsible person for Social Security)
	Cultural	No practical implementation of the corporate philosophy	[OHM] is part of the CR-strategy: employees. Does it reach me in everyday life if I ground it now just on how the CR-representative approaches me or the other way round? Then, I have to say: felt not at all. (CU210, company, employee of the human resources department with responsibility for OHM)

### Strategic issues in the interface between OHM and CSR

Participants reported that a long-term corporate strategy regarding the interface between OHM and CSR has yet to be established in most companies, but many expressed a desire that a plan of action implementing such an alignment should be set up. At present, however, participants reported that the connection between OHM and CSR is limited at the strategic level, and primarily achieved via internal standards or a company’s code of conduct.

Participants reported that a key challenge to the closer interface of OHM and CSR at the strategic level was the current difficulties in articulating the underlying ethical values relevant to both OHM and CSR. This was regarded as a problem for intra-corporation communication, and participants expressed the desire that these issues should be examined more often and more clearly in scholarship. The current divergent focus of OHM and CSR was also noted. Company representatives, in particular, emphasised that health activities were exclusively aimed at their own employees, whereas CSR encompassed a wider group of stakeholders outside the corporation. Furthermore, participants felt that health issues as well as social issues, such as sustainability and environmental protection, needed to be addressed and promoted by society as a whole and not by individual companies. Nevertheless, participants called for a framework that would enable companies to fulfil their social, environmental and health responsibilities—which participants endorsed despite their concerns—without being severely economically damaged. How this framework should look like and whether it would best be implemented through mandatory rules and regulations or by voluntary commitments and participation in networks that provide a certain infrastructure was considered open for discussion.

### Structural issues in the interface of OHM and CSR

Participants reported several ways in which companies currently structurally facilitate the interface between OHM and CSR. It was noted that the responsibility for managing OHM and CSR is often held by the same person or department in many companies. Even in companies that allocated the responsibility for OHM and CSR to different departments, participants described increasing collaboration between the two activities, for example, through the one-off or permanent participation of a representative from one department on the other department’s committee. Participants emphasised that this collaboration went in both directions and was often initiated for practical reasons, such as gaining a better understanding of the other department’s concerns and targets, facilitating more fluent communication within the company, and providing more comprehensive information for the public. Participants also noted that this structural overlapping of OHM and CSR sometimes manifested in joint activities, such as combining corporate sports activities (e.g., a company run) with CSR projects (e.g., donation to a charitable project).

However, participants also identified several structural barriers that currently inhibit the interface between OHM and CSR in many companies. Participants repeatedly emphasised that companies frequently lack the resources (both financial and knowledge) to support such interfaces, and that potential interactions depend on the company’s current economic situation. Furthermore, participants identified some more profound structural challenges that currently impede meaningful interaction between OHM and CSR. In some companies, such interfaces are currently not feasible because either their OHM or CSR department has yet to be fully developed. In other companies, OHM and CSR are undertaken at entirely different operational levels. For example, it was reported that OHM, due to its close link to legislation and the national social system, was often organised locally or regionally, while CSR is managed by a central department for all of the group’s companies.

### Cultural issues in the interface of OHM and CSR

Participants highlighted the critical role that a company’s philosophy, values, and corporate culture plays in health promotion, and noted that cultural factors could positively or negatively affect employees and their health. Participants felt that health promotion not only must take into account a company’s philosophy and culture, but can only be as successful as the corporate culture allows it to be. OHM was typically perceived by participants as falling under the broad definition of ‘social responsibility’ simply because health issues *are* a company’s social responsibility. Participants noted that acknowledgement that health is part of a company’s social responsibility is evident in discussions around work-life-balance, personal development opportunities and also in traditional occupational health and safety. It was reported that in circumstances where a ‘split’ is present between the corporate culture and the employee’s own values, employees might experience psychological strain and moral distress—for example, when employees disagree with the aims and practices of their company (e.g., selling insurance or subscriptions to vulnerable people in an aggressive way), or where diligent employees are forced to rush their work due to measures that the company has implemented in a bid to maximise output. Participants reported that the practical implementation of corporate philosophy is a key cultural barrier to interfaces between OHM and CSR. Although existing guidelines and companies’ philosophies often already connect OHM and CSR, they are currently not embraced—or ‘lived’—by the employees and managers.

## Discussion

To our knowledge, this is the first study to examine key stakeholders’ views and experiences regarding the interfaces between OHM and CSR in German companies. This study has identified several areas in which OHM and CSR are already interacting at the strategic, structural and cultural level, but it has also highlighted several barriers that undermine a more meaningful interaction.

Although the design and depth of any existing interface between OHM and CSR varied significantly among companies, certain common approaches were observed. These approaches included an overlap of responsibilities or joint activities, or a shared approach to OHM and CSR via social responsibility and a company’s corporate culture. Hence, there already appears to be significant overlap in the focus of OHM and CSR, at the structural, strategic and cultural levels in many German companies. The potential is there, both in theory and in practice, to bring OHM and CSR closer together and to start considering systematic approaches to make use of this overlap. Indeed, several examples were reported that showed that setting up interfaces takes surprisingly little effort, and for most participants, the envisioned synergies between the two fields outweighed any costs. At the same time, however, our findings support the view of Monachino and Moreira that “CSR health-related activities are generally pictured as punctual activities” [22]. At present, companies either extend their health circles or allow the responsibilities of individual managers to overlap, but few combine OHM and CSR systematically, across divisions or as part of a company-wide strategy.

### The interface between OHM and CSR

This study has identified a number of existing, overlapping values in OHM and CSR. First and foremost was the attribution of responsibility for health in the company context [40], that provide strong incentives for better integration between the two managerial branches. Moreover, the study also revealed and confirmed other ethical values that could overlap, including voluntariness and autonomy, privacy, distributive justice and issues around stigmatisation and discrimination (for the theoretical underpinning of ethical issues in WHP and OHM see: [41]). Overall, therefore, there is the potential for an improved joint, systematic consideration of ethical values within an enterprise. For example, a look at the company’s OHM activities may reveal that these mainly promote unsustainable activities, such as motor-biking or diving trips. In such cases, CSR and one of its core values—responsibility for the environment—could function as a corrective for OHM [42, 43]. CSR could also incorporate health actions, such as ‘bike to work’ or e-bike leasing schemes for employees, into its ecological/environmental dimension and its sustainability strategy where applicable. Suggestions in this direction can be found, e.g., in the ISO 26000 [44].

Interestingly, all participants in our study perceived OHM as subordinate to CSR when considering overlapping values and interactions, rather than the other way around. This outcome is perhaps unsurprising since OHM covers a much narrower field. Following Matten and Moon’s concept of implicit and explicit CSR [45], OHM could be seem to be a part of ‘implicit’ CSR given its connection to rule and regulations; while activities and that deliberative, voluntary, and exceed legal requirements are a part of `explicit´ CSR. Nevertheless, it has been recommended that in circumstances where CSR only vaguely or marginally covers health concerns, OHM may help to fill significant gaps and improve companies’ social impact and ‘explicit’ CSR ‘performance’ [46]. However, with many participants regarding CSR as the broader concept, it may be challenging to establish interfaces between OHM and CSR, both in theory and practice, in which OHM is maintained as a proper management system and not demoted to a structural component of CSR’s social dimension. Nevertheless, it is recommended that OHM and CSR are synthesised in the future to utilise the synergy between initiatives and goals, and to reap the benefits of considering social responsibility and health as two sides of the same coin.

### Implications for future practice and research

Regarding the structural and strategic interfaces between OHM and CSR, the findings indicate that two aspects of a company’s structure are critical. First, OHM and CSR staff are often located in the same department and/or report to the same manager. More research is necessary to examine the implications that may ensue from integrating WHP into one managerial branch as opposed to activities being split across different operational levels. Second, OHM and CSR stakeholders cannot be as clearly distinguished as some participants appeared to assume. A company’s own employees and the employees of their supply chain, are the beneficiaries of OHM and CSR. Codes of conduct regarding health and safety standards along the supply chain are among the more prominent areas in which the stakeholders of both management branches overlap [47]. Future research should investigate how companies may be encouraged to see ‘the big picture’ regarding their health-related activities and decisions and how they might collaborate with business partners and non-business institutions to improve employees’ health. In practice, CSR could be utilised as a corrective to OHM, which might focus too narrowly on certain groups or conditions or disregard the social context of health. Additionally, OHM could serve to deepen and extend (explicit) CSR commitments that companies have chosen to honour by adding or widening health-focussed activities.

Finally, the frequent lack of appropriate internal company structures and gaps in knowledge indicate that both OHM (and sometimes even legally mandated occupational health and safety) and CSR are neglected in some companies [21]. From a legal perspective, this amounts to a call for improved monitoring and enforcement standards. Regarding voluntary OHM and CSR activities, the development of programmes and initiatives targeted towards companies’ specific needs is essential, in addition to achieving better awareness of behaviour- and system-oriented prevention and a shared responsibility for health. These findings need to be taken into account in future studies and for designing future measures to improve health in the company context and beyond.

### Strengths and limitations

In this study in-depth interviews were conducted with a wide range of experts, who have experience with OHM and CSR in Germany. The study has captured critical aspects of reality as viewed from different stakeholders’ perspectives. However, it is possible that some key stakeholder groups were not sufficiently represented in the study. In addition, the latest amendment to German legislation that requires certifications of certain health-promoting services to maintain tax benefits might influence companies’ willingness to offer these services in the future. Furthermore, while many companies in the sample operate across Europe or worldwide, the central perspective came from German companies, their industrial partners and other institutions, as well as organisations. Therefore, the results may not adequately reflect the circumstances and conditions in other industrialised nations. No key distinctions could be identified in our study regarding diverse business sectors, which may suggest that the project’s results may have relevance for companies in all sectors. However, further research is necessary to investigate the differences between sectors regarding the overlaps and possible interaction between OMH and CSR. Finally, the study focused on in-house operations. Future research is necessary to determine the roles and responsibilities that companies could or should have in the traditionally state-dominated sphere of health promotion and health literacy in society at large [48, 49].

## Conclusion

Employers’ efforts to improve their employees’ health have increased in recent years. This study has found widespread overlap of CSR and OHM in German companies, but also important barriers and obstacles to closer integration that should not be underestimated. Although legal reforms and competitive advantages have been important drivers of such synergies between CSR and OHM, they do not seem to be sufficient by themselves to bridge management branches and merge different sets of values. To support joint approaches, it would be helpful to use a catalogue of criteria that companies can use for self-assessment or the assessment of potential industrial partners regarding CSR, OHM and their potential interfaces [reference by the authors]. Another direction would be to set up an international (e.g., European-wide) ‘health seal’ that labels a company’s occupational, social and environmental activities for its employees’, consumers’, suppliers’ and business partners’ health.

### Supplementary Information

**Additional file 1.** Interview guides of the multi-centre study on OHM and CSR in Germany. The file presents the six different interview guides that were developed for the multi-centre study on OHM and CSR in Germany and used to collect the data that are partially presented in this publication.

## Data Availability

The datasets used and analysed during the current study are available from the corresponding author on reasonable request.

## Appendix

**Table 3:** Coding Frame

Node	Main Category	Subcategory	Definition	Coding Rules
Existing interfaces & Interfaces that should be established	Operational	Joint actions	Example(s) for actions are given where OHM/ WHP and CSR are actively involved.	Punctual activities/ actions in contrast to a long-term strategy Excluding joint work on standards and certification
		Overlap of responsibilities a) in the same department b) for the same person	a) In the organisation chart (formal or informal), OHM/ WHP and CSR are attributed to the same department, but not the same person. b) One person is responsible for OHM/ WHP and CSR.	a) Explicit reference to one department responsible b) explicit reference to one single person responsible
		Extension of the health circle or CSR-board	At least one person of the other department is part of the health circle or CSR-board respectively.	Health circle or CSR-board are mentioned explicitly.
	Strategic	Standards and certification	CSR and OHM/ WHP both contribute to standards and certifications.	Standards and/ or certifications are mentioned explicitly. Contribution can be everything from a constant collaboration to punctual data interchange.
		In general	Long-term corporate alignment of CSR and OHM/ WHP, with or without a written plan of action	Strategy is mentioned explicitly or paraphrased according to the definition. Strategy other than standards and certifications
	Cultural	Corporate philosophy	A company’s value system and overall attitude towards an interplay between OHM/ WHP and CSR that goes beyond the question of responsibility.	The corporate philosophy is mentioned explicitly or paraphrased according to the definition. No strategic alignment, e.g. no plan of action, but possibly a written value statement
		Health as part of the leading principle ‘social responsibility‘	OHM/ WHP are considered to be a component or integral part of a company’s “social responsibility”. In this context, social responsibility is addressed as a fundamental value and not primarily a strategy.	“Social responsibility” or “corporate responsibility” is mentioned explicitly as a motive for or root of OHM/ WHP. No strategic alignment Focus on social/ corporate responsibility and not a company’s overall philosophy
Open concerns & Reasons for no existing/ planned interface	Operational	Lack of resources	Resources such as time, money or personnel are mentioned as concerns or reasons against an interplay.	Resources other than knowledge are listed.
		No appropriate internal structures and/ or knowledge in the company	Internal structures on the side of CSR and/ or OHM/ WHP are non-existent and/ or knowledge regarding at least one of the topics is missing.	Internal structures and/ or knowledge are mentioned explicitly.
		Location of OHM and CSR at different operational levels	The internal structures exist, but CSR and OHM/ WHP are operated and organised from different company levels.	Related to organisation chart/ company structure
	Strategic	Lack of overlapping stakeholders	The addressees of OHM/ WHP and CSR do not overlap. Therefore, also the management systems themselves cannot interplay.	Related to the persons/ groups affected by CSR and OHM/ WHP
		OHM not primarily a company task	OHM, especially non-legally mandatory health promotion is not primarily a task for companies. In contrast, public institutions, state regulations and other macrosocial structures that transcend a single company’s sphere of influence are responsible for providing a framework.	Macrosocial structures (‘the big picture’) are mentioned explicitly.
	Cultural	No practical implementation of the corporate philosophy	Corporate philosophy, i.e. value system or statement emphasising the interplay exists in theory/ on paper. However, it differs from the values that are held up and lived in the company.	Discrepancy between two value systems/ philosophies, lived and written, is explained.

## Abbreviations

CSR: Corporate Social Responsibility
OHM: Occupational Health Management
WHP: Workplace health promotion
