# Supplementary material for: Interfaces of occupational health management and corporate social responsibility: a multi-centre qualitative study from Germany
Source: BMC Public Health. 2021 Jun 2;21:1042. doi: 10.1186/s12889-021-11016-z (PMC8173898; doi:10.1186/s12889-021-11016-z)
Supplement: Supplementary file 1 — Additional file 1. Interview guides of the multi-centre study on OHM and CSR in Germany. The file presents the six different interview guides that were developed for the multi-centre study on OHM and CSR in Germany and used to collect the data that are partially presented in this publication. [file 12889_2021_11016_MOESM1_ESM.pdf]

Interview guides of the multi-centre study on OHM and CSR in Germany

Please note: The original language of the interview guides is German.

Overview:

(1) Companies – long version

(2) Companies – short version

(3) Business partners – long version

(4) Business partners – short version

(5) Non-business partners – long version

(6) Non-business partners – short version

(1) Companies – long version

1.	Introduction	
	Category	*Question*
1.1	Personal introduction and background information on the interview	First of all, thank you very much for your readiness to take part in this interview. My name is [researcher's name] and I am a research associate in the project [XXX]. This is the acronym for [XXX]. The project aims at broadening occupational health management in organisations by involving business partners and consumers. Therefore, our project is also linked to the field of CSR/ social responsibility. In a first step we conduct interviews with experts of companies to gain a deeper understanding of their perspective.
1.2	Introduction to the company	Please introduce yourself and your company briefly.

2.	OHM – status quo [alternatively No. 3]	
2.1.1	Working conditions	a) In which units do you seem to have particularly healthy employees?<br>b) In which units do your employees seem to be increasingly ill?
2.1.2		How do explain these phenomena?
2.2.1	Understanding of OHM	a) What do you understand by 'OHM'?<br>b) Where does OHM play a role in your company?
2.2.2		What does your company motivate to offer and carry out OHM measures?
2.3.1	OHM offer	Which OHM measures do you offer your employees?
2.3.2		How are these perceived by your employees?
2.3.3.		Do group specific offers exist?
2.3.4		Do employees have the opportunity to influence the OHM offer?
2.4.1	OHM as a corporate strategy	Would you rather assign OHM in your company to the corporate strategy or the corporate culture?<br>Or both?
2.4.2		a) Is there a corporate consensus on how OHM optimally should look like in your company?<br>b) If not: What dissents exists?
2.4.3		What medium- and long-term developments does your company pursue in the field of OHM?
2.5.1	OHM with regard to business partners	a) Does your OHM also concern the employees of your business partners?

		b) If so, in what way?
2.5.2		a) Do the criteria you choose your business partners with also include OHM activities?<br>b) If so: What do you particularly value?
2.5.3		a) Is there a joint exchange or joint measures with your business partners in the field of OHM?<br>b) Is there an exchange with NGOs or government bodies?
2.5.4		a) What would have to happen to end the cooperation with one of your business partners?<br>b) Has something like this already happened?
2.6.1	Legal or ethical assumption of responsibility?	Should OHM measures be legally mandatory, or should companies be free to decide on the introduction and implementation of OHM?
2.6.2		In which way should OHM measures spread:<br>a) purely through information?<br>b) via decision influencing, i.e. the decision architecture?<br>c) by setting incentives?<br>d) via company internal regulations?<br>e) via SoPs (statements of position)?<br>f) via directives or laws?<br>g) via taxes?<br>h) via other harsh sanctions?
2.7.1	Ethical Perspective on OHM	How do you evaluate OHM from an ethical perspective?
2.7.2		In your opinion, are there besides economic also ethical reasons for implementing and maintaining OHM?

2.7.3		Do you think that OHM opens up better solutions for existing ethical problems?
2.7.4		Do additional ethical problems arise by OHM?
2.7.5		Do you believe that one can solve ethical problems with OHM?
2.8.1	Communication of OHM	How are OHM activities communicated externally and internally?
2.8.2		What do you think: How do your customers inform themselves about your OHM activities?
2.8.3		Please estimate how much value your customers place on information about OHM.
3.	CSR – status quo [alternatively No. 2]	
3.1.1	Understanding of CSR	a) What do you understand by 'CSR'?<br>b) What role does CSR play in your company?
3.1.2		Please tell me about particularly positive measures/ actions/ highlights of your company in the field of CSR.
3.1.3		For which topics is the CSR-work particularly important in your company?
3.1.4		What does your company motivate to pursue CSR measures?
3.2.1	Employees' influence on CSR measures	Are there CSR measures in which employees can participate?
3.2.2		How is the offer perceived by employees?
3.2.3		a) Do employees have the opportunity to influence the CSR offer?<br>b) If so, in which way?

3.3.1	CSR as part of the corporate culture	Would you rather assign CSR in your company to the corporate strategy or the corporate culture?<br>Or both?
3.3.2		a) Is there a corporate consensus on how CSR optimally should look like in your company?<br>b) If not: What dissents exists?
3.3.3		What medium- and long-term developments does your company pursue in the field of CSR?
3.4.1	CSR with regard to business partners	a) Do your CSR activities also have an impact on your business partners?<br>b) If so, in what way?
3.4.2		a) Do the criteria you choose your business partners with also include CSR activities?<br>b) If so: What do you particularly value?
3.4.3		a) Is there a joint exchange or joint measures with your business partners in the field of CSR?<br>b) Is there an exchange with NGOs or government bodies?
3.4.4		a) What would have to happen to end the cooperation with one of your business partners?<br>b) Has something like this already happened?
3.5.1	Legal or ethical assumption of responsibility?	Should CSR measures be legally mandatory, or should companies be free to decide on the introduction and implementation of CS?
3.5.2		In which way should CSR measures spread:<br>a) purely through information?

		b) via decision influencing, i.e. the decision architecture?<br>c) by setting incentives?<br>d) via company internal regulations?<br>e) via SoPs (statements of position)?<br>f) via directives or laws?<br>g) via taxes?<br>h) via other harsh sanctions?
3.6.1	Ethical Evaluation of CSR	How do you evaluate CSR from an ethical perspective?
3.6.2		In your opinion, are there besides economic also ethical reasons for implementing and maintaining CSR?
3.6.3		Do you think that CSR opens up better solutions for existing ethical problems?
3.6.4		Do additional ethical problems arise by CSR?
3.6.5		Do you believe that one can solve business ethical problems with CSR?
3.7.1	Communication of CSR	How are your CSR activities communicated externally and internally?
3.7.2		What do you think: How do your customers inform themselves about your CSR activities?
3.7.3		Please estimate how much value your customers place on information about the topic of CSR.
4.	Interface of CSR and OHM	
4.1.1	Corporate Responsibility	On a scale of 1 to 5, how much responsibility for employee health would you attribute to companies? Why?

4.1.2		a) Do you see yourself as having a duty towards the health of your business partners' employees?<br>b) How much responsibility would you attribute to yourself on a scale of 1 to 5?
4.1.3		In your opinion, are there social obligations between you and your business partners?
4.2.1	Link between CSR and OHM	Are the topics of health promotion and social responsibility addressed jointly in your company?
4.2.2		If yes (alternatively 4.2.3):<br>a) How is this achieved and what are the reasons?<br>b) Is there a strategy for this?<br>c) What reasons, beyond the economic and operational, play a role in this?<br>d) Should the cooperation between CSR and OHM be expanded in the future?
4.2.3		If no (alternatively 4.2.2):<br>a) Should OHM and CSR be addressed jointly?<br>b) Do you think there are good reasons (also beyond the economic and operational) to address OHM and CSR jointly?
5.	Concluding questions	
5.1.1	Beneficial and detrimental	To conclude, I would like to ask you as a kind of summary what, in your view, are beneficial and detrimental conditions for OHM.

5.1.2	conditions for OHM and CSR	What are, in your view, beneficial and detrimental conditions for the assumption of social responsibility by the company?
5.2	Further remarks	Is there something from the fields of OHM/CSR or business partners that has not been asked but seems relevant to you?

(2) Companies – short version

1.	Context – Organisation in general and the position of the interview partner within the organisation	
-		One remark before we start: There are no right or wrong answers. It is simply a matter of depicting the current status quo. This is then analysed anonymously.
1.1	Introduction to the company	Please introduce yourself and your company briefly.<br>a) Kind of products/ services<br>b) Conditions regarding business partners<br>c) Conditions regarding customers
1.2.1		What is your position in the organisation?
1.2.2		What are your tasks and what decision-making authority do you have?
1.2.3		What is your responsibility in the field of OHM/ CSR?
2.	OHM – status quo [alternatively No. 3]	
2.1.1	Introduction to OHM via working conditions	Where are the working conditions in your company particularly good?
2.1.2		In which areas is the workload particularly high?
2.2	Understanding of OHM	a) What do you understand by 'OHM'?<br>b) Where does OHM play a role in your company?

2.3.1	Structural framework for OHM in the organisation	How is OHM structurally anchored in the company?
2.3.2		Who is responsible for this?
2.3.3		Is there a special group or specific persons who take care of it?
2.3.4		Is there a budget for it?
2.4.1	OHM offer resp. measures	Are measures carried out to influence the working conditions in the company?
2.4.2		What kind of measures do you offer to employees?
2.4.3		Do they take place during working hours?
2.5.1	Access to OHM and involvement of employees regarding the design of offers	For whom are these offers?
2.5.2		Are there also measures for specific groups of employees?
2.5.3		Are employees involved in the preparation of the offer of OHM measures? If so, how?
2.5.4		How is the offer accepted resp. perceived by the employees?
2.6.1	OHM as a corporate strategy	Would you say that OHM is strategically anchored in the corporate guidelines?
2.6.2		[If yes:] Do the employees of your company know that the topic of health is of importance to the company?
2.6.3		What medium- and long-term developments does your company pursue in the field of OHM?
2.6.4		What would you say, how relevant is OHM for top management?
2.6.5		Why does your company carry out OHM measures?

2.7.	Beneficial and detrimental conditions for OHM	In your view, what are beneficial and detrimental conditions for OHM?
2.8.1	Communication of OHM	(How) Is your OHM action communicated internally (to employees)?
2.8.2		(How) Is your OHM action communicated externally (to customers and business partners)?
3.	CSR – status quo [alternatively No. 2]	
3.1.1	Understanding of CSR and concrete actions	What do you understand by 'CSR'?
3.1.2		Please tell me about particularly positive measures/ actions/ highlights of your company in the field of CSR.
3.1.3		For which topics is the CSR-work particularly important in your company?
3.1.4		How does your company fulfil its social responsibility in general?
3.2.1	Structural framework for CSR	How is CSR structurally anchored in the company?
3.2.2		Which person/ group is responsible for this?
3.2.3		Is there a budget for it?
3.3.1	CSR as part of the corporate culture	Would you rather assign CSR in your company to the corporate strategy or the corporate culture? Or both?
3.3.2		Do the employees of your company know about the value of CSR in your company?

3.3.3		What medium- and long-term developments does your company pursue in the field of CSR?
3.3.4		How relevant is CSR for top management of your company?
3.3.5		What is the motivation of your company to pursue CSR measures?
3.4.1	Beneficial and detrimental conditions for CSR	In your view, what are beneficial and detrimental conditions for CSR?
3.4.2	Communication of CSR	How are your CSR activities communicated externally and internally?
4.	Business partners – status quo	
4.1.1	Criteria for the selection of business partners	With regard to company's suppliers and business partners: On the basis of which criteria is decided with whom to cooperate?
4.1.2		[If not mentioned directly:] To what extent do working conditions at the suppliers play a role?
4.2	Influence on the business partners' working conditions	In your opinion: Dou your own guidelines for the selection of suppliers influence their working conditions?
4.3.1	Collaboration regarding working conditions	Does a collaboration or exchange with your suppliers and business partners exist regarding the design of working conditions?
4.3.2		[If so:] How does the collaboration look like in concrete terms?

4.4.1	Involvement of other stakeholders regarding OHM	Do you use support of external organisations (e.g. health insurances, networks) regarding the design of your OHM?
4.4.2		Do you include feedback from customers/ suppliers/ business partners/ employees in your OHM?
5.	Interface of CSR and OHM	
5.1.1	Corporate responsibility for employees' health	What would you say: Who is responsible for the health of employees?
5.1.2		How much responsibility for employee health would you attribute to companies in general, from 0% to 100%? Why?
5.1.3		And what about your own company?
5.2	Corporate responsibility for business partners' employees	Do you see yourself as having a duty towards your business partners' employees?
5.3.1	Link between CSR and OHM	Are the topics of health promotion and social responsibility addressed jointly in your company?
5.3.2		If yes (alternatively 5.3.3):<br>a) How is this achieved and what are the reasons?<br>b) Is there a strategy for this?
5.3.3		If no (alternatively 5.3.2):<br>a) Do you think there are good reasons (also beyond the economic and operational) to address OHM and CSR jointly?<br>b) Why (not)?
6.	Concluding question	

		Is there something from the fields of OHM/CSR or business partners that has not been asked but seems relevant to you?

(3) Business partners – long version

1.	Introduction	
1.1	Personal introduction and background information on the interview	First of all, thank you very much for your readiness to take part in this interview. My name is [researcher's name] and I am a research associate in the project [XXX]. This is the acronym for [XXX]. The project aims at broadening occupational health management in organisations by involving business partners and consumers. Therefore, our project is also linked to the field of CSR/ social responsibility. In a first step we conduct interviews with experts of companies to gain a deeper understanding of their perspective.
1.2	Introduction to the company	Please introduce yourself and your company/ your organisation briefly.
2.	Cross-link with the company X [a company already interviewed with interview guide (1)]	
2.1.1	The business partner's cooperation policy	What is your relationship to company X?
2.1.2		Do you cooperate with companies on a permanent basis or within the scope of projects?
2.1.3		Why do you cooperate with company X?
2.1.4		a) What do you appreciate about this company?<br>b) What do you see rather critically?

2.2.1	Values	a) What is important to you in your company/organisation when it comes to the working atmosphere?<br>b) Which 'soft' factors are important to you?
2.2.2		What does a good collaboration with other companies look like for you in general?
2.2.3		a) What would have to happen to end the cooperation with one of your business partners?<br>b) Has something like this already happened?
3.	OHM – status quo [alternatively No. 4]	
3.1.1	Existing standards	[If applicable:] What standards and measures make up your OHM?
3.1.2		What do you put emphasis on?
3.2.1	Responsibility in the field of OHM	[Either:]<br>Who is responsible for the employees' health in your company?<br>[Or more generally:]<br>Who is responsible for the employees' health in supplier companies?
3.2.2		What kind of work/ employee management do you associate with company X?
3.2.3		[Either:]<br>To your knowledge: Does company X take responsibility for their employees, especially in the field of occupational safety and health?<br>[Or more generally:]

		Who takes responsibility for which areas, especially regarding employees?
3.2.4		[If applicable:] Do responsibilities overlap in the field of OHM between your company/ organisation and company X?
3.3.1	Importance of OHM	[If applicable:] How important do you consider OHM to be in your company/ organisation?
3.3.2		And how important in company X?
3.4	Consumers' role	How do you estimate consumers' interest in this topic?
3.5.1	Legal or ethical assumption of responsibility?	Should OHM measures be legally mandatory, or should companies be free to decide on the introduction and implementation of OHM?
3.5.2		In which way should OHM measures spread:<br>a) purely through information?<br>b) via decision influencing, i.e. the decision architecture?<br>c) by setting incentives?<br>d) via company internal regulations?<br>e) via SoPs (statements of position)?<br>f) via directives or laws?<br>g) via taxes?<br>h) via other harsh sanctions?
3.6.1	Ethical Perspective on OHM	How do you evaluate OHM from an ethical perspective?
3.6.2		In your opinion, are there besides economic also ethical reasons for implementing and maintaining OHM?
3.6.3		Do you think that OHM opens up better solutions for existing ethical problems?

3.6.4		Do additional ethical problems arise by OHM?
3.6.5		Do you believe that one can solve ethical problems with OHM?
4.	CSR – status quo [alternatively No. 3]	
4.1.1	Understanding of CSR	What do you understand by 'CSR'?
4.1.2		Is your company/ organisation active in this field?
4.1.3		[If applicable:] What measures can you emphasise in particular?
4.1.4		Would you consider social responsibility to be an essential part of your corporate/ organisational culture?
4.1.5		How important would you rate CSR in company X?
4.1.6		Which CSR measures do you view positively, which critically?
4.2	Consumers' role	How do you estimate consumers' interest in this topic?
4.3.1	Legal or ethical assumption of responsibility?	Should CSR measures be legally mandatory, or should companies be free to decide on the introduction and implementation of CS?
4.3.2		In which way should CSR measures spread:<br>a) purely through information?<br>b) via decision influencing, i.e. the decision architecture?<br>c) by setting incentives?<br>d) via company internal regulations?<br>e) via SoPs (statements of position)?<br>f) via directives or laws?<br>g) via taxes?

		h) via other harsh sanctions?
4.4.1	Ethical Evaluation of CSR	How do you evaluate CSR from an ethical perspective?
4.4.2		In your opinion, are there besides economic also ethical reasons for implementing and maintaining CSR?
4.4.3		Do you think that CSR opens up better solutions for existing ethical problems?
4.4.4		Do additional ethical problems arise by CSR?
4.4.5		Do you believe that one can solve business ethical problems with CSR?
5.	Interface of CSR and OHM	
5.1.1	Link between CSR and OHM	Are the topics of health promotion and social responsibility addressed jointly in your company/organisation?
5.1.2		If yes (alternatively 5.1.3):<br>a) Can you name concrete measures at this interface?<br>b) What is it like with company X?
5.1.3		If no (alternatively 4.2.2) or if 5.1.1 is not applicable:<br>Are there companies where you recognize an interface between OHM and CSR?<br>If yes: Do you know about concrete measures of companies where both fields interact?<br>If no: Should OHM and CSR be addressed jointly??
6.	Concluding questions	
6.1.1		How could the OHM of your business partner (i.e. company X) benefit from a cooperation?

6.1.2	Widening the scope to the supply chain	Do you think that good or bad practices in the field of CSR have an impact on customers' evaluation of your company/ organisation?
6.1.3		And what does that look like in the field of OHM?
6.2	Further remarks	Is there something from the fields of OHM/CSR or business partners that has not been asked but seems relevant to you?

(4) Business partners – short version

1.	Context – Industry sector & Specifics of the industry sector regarding OHM<br>Position of the interview partner	
-		One remark before we start: There are no right or wrong answers. It is simply a matter of depicting the current status quo. This is then analysed anonymously.
1.1.1	Position of the interview partner	In which field do you work or which industry sector do you represent?
1.1.2		What are your tasks?
1.2.1	Conditions concerning stakeholders	What types of companies can typically be found in this industry sector?
1.2.2		With which companies do you typically cooperate?
1.2.3		What kind of contact do companies in this industry sector typically have with end consumers?
2.	OHM – status quo	
2.1.1		With regard to working conditions in your industry sector: Where do you see challenges?

2.1.2	Introduction to OHM via working conditions	And in which areas do you already see very good working conditions?
2.2.1	Industry sector specific OHM	What specifics occur in your industry sector with regard to OHM?
2.2.2		How is OHM implemented in your industry sector?
2.3.	Beneficial and detrimental conditions for OHM	In your view, what are beneficial and detrimental conditions for OHM?
3.	Business partners – status quo	
3.1.1	Criteria for the selection of business partners	With regard to company's suppliers and business partners: Are there industry sector specific criteria that are used to decide with whom companies cooperate?
3.1.2		[If not mentioned directly:] To what extent do working conditions at the suppliers play a role?
3.2	Influence on the business partners' working conditions	In your opinion: Do companies influence your working conditions, e.g., through their own guidelines regarding the selection of suppliers, external service providers and other business partners?
3.3.1	Collaboration regarding working conditions	Does a collaboration or exchange of companies regarding the design of working conditions take place in your industry sector?
3.3.2		[If so:] How does the collaboration look like in concrete terms?

4.	Interface of CSR and OHM	
4.1.1	Corporate responsibility for employees’ health	How much responsibility for employee health would you attribute to companies in general, from 0% to 100%? Why?
4.1.2		What do you understand by ‘CSR’ in general?
4.2.1	Link between CSR and OHM	Are the topics of health promotion and social responsibility addressed jointly in your industry sector?
4.2.2		If yes (alternatively 5.3.3):<br>a) How is this achieved and what are the reasons?<br>b) Is there a strategy for this?
4.3	Beneficial and detrimental conditions for CSR	In your view, what are beneficial and detrimental conditions for the assumption of social responsibility by companies?
5.	Concluding question	
		Is there something from the fields of OHM/CSR or business partners that has not been asked but seems relevant to you?

(5) Non-business partners – long version

1.	Introduction	
1.1	Personal introduction and background information on the interview	First of all, thank you very much for your readiness to take part in this interview. My name is [researcher’s name] and I am a research associate in the project [XXX]. This is the acronym for [XXX]. The project aims at broadening occupational health management in organisations by involving business partners and consumers. Therefore, our project is also linked to the field of CSR/ social

		responsibility. In a first step we conduct interviews with experts of companies to gain a deeper understanding of their perspective.
1.2	Introduction to the industry sector	Please introduce yourself and the industry sector you represent briefly.
2.	OHM – status quo [alternatively No. 3]	
2.1.1	Understanding of OHM	What do you understand by 'OHM'?
2.1.2		Where does OHM play a major role in your industry sector?
2.2.1	Typical OHM topics of the industry sector	What typical OHM topics are you dealing with in your industry sector?
2.2.2		Are there typical diseases/ problems that concern you?
2.2.3.		In which areas within your industry sector is the workload particularly high?
2.2.4		Are certain measures offered for employees that are typical for the industry sector?<br>If yes: How is this offer perceived?
2.2.5		Without having to mention names: Can you think of pioneers or latecomers of OHM in your industry sector? What distinguishes them?
2.3.1	OHM as a corporate strategy	Would you say that OHM is a strategic element in your industry sector?
2.3.2		a) Is there a consensus on how OHM optimally should look like in your industry sector?<br>b) If not: Does a minimal consensus exist at least?

2.4.1	Industry-wide influence	a) Do you have influence on the OHM of companies in your industry sector?<br>b) If so, how is this influence shaped? And can you influence company internal OHM strategies?
2.4.2		Are there specialised contact persons in the field of OHM in your industry sector who companies can contact?
2.4.3		Is there a contact point in your industry sector outside of companies that employees can turn to with questions, complaints and suggestions regarding OHM?
2.4.4		a) Is a budget provided to deal with problems of OHM in your industry sector?<br>b) If so: Where do these grants come from?
2.5.1	Legal or ethical assumption of responsibility?	Should OHM measures be legally mandatory, or should companies be free to decide on the introduction and implementation of OHM?
2.5.2		In which way should OHM measures spread:<br>a) purely through information?<br>b) via decision influencing, i.e. the decision architecture?<br>c) by setting incentives?<br>d) via company internal regulations?<br>e) via SoPs (statements of position)?<br>f) via directives or laws?<br>g) via taxes?<br>h) via other harsh sanctions?
2.6.1		How do you evaluate OHM from an ethical perspective?

2.6.2	Ethical Perspective on OHM	In your opinion, are there besides economic also ethical reasons for implementing and maintaining OHM?
2.6.3		Do you think that OHM opens up better solutions for existing ethical problems?
2.6.4		Do additional ethical problems arise by OHM?
2.6.5		Do you believe that one can solve ethical problems with OHM?
2.7.1	Communication of OHM	How are OHM activities in your industry sector typically communicated by companies internally and externally?
2.7.2		Are there ways of communication that are perceived more positively/ negatively?
2.7.3		How well informed are customers about OHM activities?
2.7.4		Please estimate how much value customers place on information about OHM.
3.	CSR – status quo [alternatively No. 2]	
3.1.1	Understanding of CSR	What do you understand by 'CSR'?
3.1.2		Where does CSR play a major role in your industry sector?
3.2.1	Typical CSR topics of the industry sector	What typical CSR topics are you dealing with in your industry sector?
3.2.2		In which areas within the industry sector is the demand for social responsibility particularly high?
3.2.3.		In which areas is the voluntarily assumed social responsibility particularly high?

3.2.4		Are certain measures offered that are typical for the industry sector and actively involve employees?<br>If yes: How is this offer perceived?
3.2.5		Without having to mention names: Can you think of pioneers or latecomers of CSR in your industry sector? What distinguishes them?
3.3.1	CSR as a corporate strategy	Would you say that CSR is a strategic element in your industry sector?
3.3.2		a) Is there a consensus on how CSR optimally should look like in your industry sector?<br>b) If not: Does a minimal consensus exist at least?
3.3.3		Are there certain standards in your industry sector by which CSR is interpreted and evaluated?
3.4.1	Industry-wide influence	a) Do you have influence on the CSR activities of companies in your industry sector?<br>b) If so, how is this influence shaped?
3.4.2		Are there specialised contact persons in the field of CSR in your industry sector who companies can contact?
3.4.3		Is there a contact point in your industry sector outside of companies that employees can turn to with questions, complaints and suggestions regarding CSR?
3.4.4		a) Is a budget provided to deal with problems of CSR in your industry sector?<br>b) If so: Where do these grants come from?

3.5.1	Legal or ethical assumption of responsibility?	Should CSR measures be legally mandatory, or should companies be free to decide on the introduction and implementation of CSR?
3.5.2		In which way should CSR measures spread:<br>a) purely through information?<br>b) via decision influencing, i.e. the decision architecture?<br>c) by setting incentives?<br>d) via company internal regulations?<br>e) via SoPs (statements of position)?<br>f) via directives or laws?<br>g) via taxes?<br>h) via other harsh sanctions?
3.6.1	Ethical Perspective on CSR	How do you evaluate CSR from an ethical perspective?
3.6.2		In your opinion, are there besides economic also ethical reasons for implementing and maintaining CSR?
3.6.3		Do you think that CSR opens up better solutions for existing ethical problems?
3.6.4		Do additional ethical problems arise by CSR?
3.6.5		Do you believe that one can solve ethical problems with CSR?
3.7.1	Communication of CSR	How are CSR activities in your industry sector typically communicated by companies internally and externally?
3.7.2		Are there ways of communication that are perceived more positively/ negatively?
3.7.3		How well informed are customers about CSR activities?

3.7.4		Please estimate how much value customers place on information about CSR.
4.	Interface of CSR and OHM	
4.1.1	Responsible parties within the industry sector	What would you say: Who is responsible for the health of employees?
4.1.2		On a scale of 1 to 5, how much responsibility for employee health would you attribute to companies? Why?
4.1.3		And how much responsibility for the health of employees in cooperating companies would you attribute to the same company, on a scale of 1 to 5? Why?
4.2.1	Link between CSR and OHM	Are the topics of health promotion and social responsibility usually addressed jointly in your industry sector?
4.2.2		If yes (alternatively 4.2.3):<br>a) How is this achieved and what are the reasons?<br>b) Is there a strategy for this?<br>c) What reasons, beyond the economic and operational, play a role in this?<br>d) Should the cooperation between CSR and OHM be expanded in the future?
4.2.3		If no (alternatively 4.2.2):<br>a) Should OHM and CSR be addressed jointly?<br>b) Do you think there are good reasons (also beyond the economic and operational) to address OHM and CSR jointly?
5.	Concluding questions	

5.1.1	Beneficial and detrimental conditions for OHM and CSR	To conclude, I would like to ask you as a kind of summary what, in your view, are beneficial and detrimental conditions for OHM.
5.1.2		What are, in your view, beneficial and detrimental conditions for the assumption of social responsibility by the company?
5.1.3		[If applicable:] What do you regard to be positive and what critical about OHM/ CSR?
5.2	Further remarks	Is there something from the fields of OHM/CSR or business partners that has not been asked but seems relevant to you?

(6) Non-business partners – short version

1.	Context – Specifics regarding OHM & position of the interview partner	
-		One remark before we start: There are no right or wrong answers. It is simply a matter of depicting the current status quo. This is then analysed anonymously.
1.1	Position of the interview partner	First of all, could you please introduce yourself and your range of activities in the field of OHM?
1.2.1	Conditions concerning stakeholders	What types of companies or organisations are you typically in contact within your work?
1.2.2		What kind of contact do you typically have in your work with end consumers (of the respective company)?
1.2.3		[If there is contact:] Do you know/ use concepts for the involvement of customers in OHM?
2.	OHM – status quo	

2.1	OHM in the present	In your experience, how (i.e. through which measures) is OHM currently implemented in organisations?
2.2.1	Introduction to OHM via working conditions	With regard to working conditions in companies: Where do you see special challenges for the health of employees?
2.2.2		And in which areas do you already see very good working conditions?
2.3	OHM in the future	In your opinion, what are the specifics of successful OHM?
2.4.	Beneficial and detrimental conditions for OHM	In your view, what are beneficial and detrimental conditions for OHM?
3.	Business partners – status quo	
3.1	Criteria for the selection of business partners	Often there are specific criteria for companies to decide with which partners or suppliers to cooperate. In your experience, to what extent do OHM or working conditions play a role?
3.2	Influence on the business partners' working conditions	In your opinion: Do companies influence suppliers', external service providers' or other business partners' working conditions, e.g., through the companies own guidelines regarding the selection of these partners?
3.3.1	Collaboration regarding working conditions	To what extent do companies cooperate with other companies or business partners regarding OHM?
3.3.2		Does a collaboration or exchange regarding the design of working conditions take place?
3.3.3		[If so:] How does the collaboration look like in concrete terms?

3.3.4		Do you know/use concepts for the involvement of several companies in OHM?
4.	Interface of CSR and OHM	
4.1.1	Corporate responsibility for employees' health	How much responsibility for employee health would you attribute to companies in general, from 0% to 100%? Why?
4.1.2		What do you understand by 'CSR' in general?
4.2.1	Link between CSR and OHM	Are the topics of health promotion and social responsibility addressed jointly in companies?
4.2.2		If yes (alternatively 5.3.3):<br>a) How is this achieved and what are the reasons?<br>b) Is there a strategy for this?
4.3	Beneficial and detrimental conditions for CSR	In your view, what are beneficial and detrimental conditions for the assumption of social responsibility by companies?
5.	Concluding question	
		Is there something from the fields of OHM/CSR or business partners that has not been asked but seems relevant to you?
